# Effect of plateletcrit and *methylenetetrahydrofolate reductase (MTHFR)* C677T genotypes on folic acid efficacy in stroke prevention

**DOI:** 10.1038/s41392-024-01817-0

**Published:** 2024-05-10

**Authors:** Yuncong Shi, Zhengzhipeng Zhang, Binyan Wang, Yu Wang, Xiangyi Kong, Yong Sun, Aimin Li, Yimin Cui, Yan Zhang, Jianping Li, Yong Huo, Hui Huang

**Affiliations:** 1 Cardiovascular Department, The Eighth Affiliated Hospital, Joint Laboratory of Guangdong-Hong Kong-Macao Universities for Nutritional Metabolism and Precise Prevention and Control of Major Chronic Diseases, Sun Yat-sen University, Shenzhen, China; 2 Shenzhen Evergreen Medical Institute, Shenzhen, China; 3 Shenzhen Tailored Medical Laboratory, Shenzhen, China; 4 Department of Cardiology, Beijing Anzhen Hospital, Capital Medical University, Beijing, China; 5 Department of Neurosurgery, The First Affiliated Hospital of Kangda College of Nanjing Medical University, Lianyungang, China; 6 Institute of Clinical Pharmacology, Peking University First Hospital, Beijing, China; 7 Department of Cardiology, Peking University First Hospital, Beijing, China

**Keywords:** Cardiology, Prognostic markers

## Abstract

Previous studies have shown that low platelet count combined with high plasma total homocysteine (tHcy) increased stroke risk and can be lowered by 73% with folic acid. However, the combined role of other platelet activation parameters and the methylenetetrahydrofolate reductase (*MTHFR*) C677T genotypes on stroke risk and folic acid treatment benefit remain to be examined. This study aimed to investigate if platelet activation parameters and *MTHFR* genotypes jointly impact folic acid treatment efficacy in first stroke prevention. Data were derived from the China Stroke Primary Prevention Trial. This study includes a total of 11,185 adult hypertensive patients with relevant platelet activation parameters and *MTHFR* genotype data. When simultaneously considering both platelet activation parameters (plateletcrit, platelet count, mean platelet volume, platelet distribution width) and *MTHFR* genotypes, patients with both low plateletcrit (Q1) and the TT genotype had the highest stroke incidence rate (5.6%) in the enalapril group. This subgroup significantly benefited from folic acid treatment, with a 66% reduction in first stroke (HR: 0.34; 95% CI: 0.14–0.82; *p* = 0.016). Consistently, the subgroup with low plateletcrit (Q1) and the CC/CT genotype also benefited from folic acid treatment (HR: 0.40; 95% CI: 0.23–0.70; *p* = 0.001). In Chinese hypertensive adults, low plateletcrit can identify those who may greatly benefit from folic acid treatment, in particular, those with the TT genotype, a subpopulation known to have the highest stroke risk.

## Introduction

About 50% of cardiovascular and cerebrovascular disease deaths are closely related to hypertension, which is one of the major culprit for death.^1^ Stroke is the most common cardiovascular event in hypertensive patients. Hyperhomocysteinemia is regarded as an important risk factor for increased stroke risk. A previous report revealed that folic acid can reduce the 21% stroke risk in Chinese hypertensive patients, mainly through reducing total homocysteine (tHcy).^2^ At present, clinical guidelines suggest that folic acid can decrease serum tHcy to a certain extent and reduce the risk of stroke.^3^ However, not all hypertensive patients with elevated tHcy will benefit from folic acid supplementation. Methylenetetrahydrofolate reductase (*MTHFR*) is a folic acid metabolism crucial factor. There is a single nucleotide gene polymorphism C677T in the *MTHFR* coding gene and mutations in this enzyme lead to decreased activity and heat resistance, resulting in increased tHcy levels.^4^ Carriers of the *MTHFR* C677T gene mutation are not sensitive to folic acid treatment due to folate utilization disorder. Huang et al. showed that hypertensive patients with the *MTHFR* C677T gene mutation caused an increased incidence of stroke due to folic acid metabolism disorder, however they did not benefit from folic acid treatment.^5^ The proportion of TT homozygous genotype population in Europe and the United States is about 11.1 to 32.2%.^6,7^ The distribution of the *MTHFR* 677 CC, CT and TT genotype in a European population was reported by Spence et al. as 40.4%, 46.6% and 13%, respectively.^8^ The prevalence of the Chinese hypertensive *MTHFR* 677 TT genotype adults is much larger than other countries. In China, there are 300 million people with hypertension and of those, about 17.9 to 25% had *MTHFR* 677 TT genotype, that is, there are about 53.7 to 75 million hypertensive people with *MTHFR* 677 TT genotype.^9,10^ A meta-analysis in the Lancet showed that the *MTHFR* 677 TT genotype compared with CC homozygotes increased the risk of stroke in Western populations without cardiovascular disease by 26%.^11^ Therefore, identifying an effective biomarker among *MTHFR* 677 TT genotype to guide folic acid treatment is important for reducing first stroke risk.

Previous studies have found that elevated tHcy can increase the production of thrombin by platelets, thereby affecting platelet aggregation and the activity of coagulation factors, accelerating platelet activation, promoting coagulation and inhibiting anticoagulation, thus increasing the risk of stroke.^12^ Platelet activation and aggregation promote the platelet vasoactive factors production, resulting in vascular endothelial cell injury and decreased vascular elasticity.^13^ It has been reported that platelet activation may be a crucial intermediate link by which hyperhomocysteinemia promotes stroke progression. However, folic acid treatment can reduce platelet activation levels. This suggests that the assessment of folic acid in the treatment of stroke based on platelet activation status may have important value.^14,15^ Studies have reported that folic acid treatment can increase tetrahydrobiopterin levels, participate in the synthesis of nitric oxide in the body, decrease superoxide anion levels, improve endothelial function, maintain the elasticity and patency of vessels, and thus reduce the risk of thrombosis.^16^ Kong et al.^17^ found that folic acid treatment reduced the 73% stroke risk in a subgroup with low platelet count (PLT) and high tHcy among Chinese hypertensive adults.^17^ Platelet activation parameters may best reflect the folic acid clinical efficacy. Nevertheless, the effect of other platelet activation parameters on the prevention of first stroke risk with folic acid treatment is unclear. A recent study identified plateletcrit (PCT) is a early-warning index for stroke.^18^ The rise in blood pressure accelerates platelet aggregation, resulting in enhanced platelet consumption and decreased PCT.^19–21^ Platelet volume related activation parameters are a series of simple and easy to measure indicators, which reflect the level of platelet activation through the platelet morphology and structure changes. In vitro studies have shown that the hypertension increases the stress on the blood vessel wall, causing mechanical damage that destroys the integrity of blood vessel endothelial cells and promotes platelet activation.^22,23^ Increased platelet activation leaded to massive platelet depletion. With time, the hematopoietic function of bone marrow underwent compensatory proliferation and accelerated megakaryocyte to product more small platelets.^19–21^ The newly generated platelets had particularly active features in terms of metabolism and enzymes, and their dense particles increase the production of thromboxane A2 and B2, thereby accelerating thrombosis.^24^

Folic acid treatment to reduce tHcy level and platelet activation while taking into account *MTHFR* C677T genotypes polymorphism is an important strategy for prevention and control of stroke in China, and it is also the most economical and effective method to curb the high incidence of stroke. At present, the combined effect and clinical significance of platelet activation parameters and *MTHFR* C677T genotypes in folic acid treatment for stroke risk prevention remain to be systematically studied. Therefore, this study aimed to identify the subpopulations who could benefit most from folic acid treatment in reducing the first stroke risk through simultaneous consideration of *MTHFR* C677T genotypes and platelet activation parameters in hypertensive patients.

## Results

Supplementary Fig. 1 showed the study flow chart. Since platelet parameters’ baseline measurements were only obtained at the Lianyungang study center of the Chinese Stroke Primary Prevention Study (CSPPT), our analysis was limited to the 15,486 participants from this center. Participants with missing data on platelet parameters or tHcy, or those on antiplatelet drugs at the trial entry were also excluded from the study. There were 11,185 patients in the study, of whom 5602 were treated with enalapril-folic acid and 5583 were only treated with enalapril. The median time of study was 4.2 years, and 385 patients occurred first strokes.

### Baseline characteristics of study participants by *MTHFR* C677T genotype

As shown in Table 1, participant baseline characteristics are presented according to *MTHFR* C677T genotype: normal (CC/CT) and mutant (TT). With the exception of blood glucose, tHcy, folic acid, and platelet distribution width (PDW), other baseline characteristics were not significantly different in patients with *MTHFR* 677 CC/CT and TT genotypes. There were significant differences in PDW in the *MTHFR* 677 CC/CT genotype group and in creatinine in the *MTHFR* 677 TT genotype group between the enalapril and enalapril-folic acid treatment groups. All other baseline characteristics were not significantly different between the treatment groups (enalapril and enalapril-folic acid). In addition, when baseline characteristics were presented according to PCT quartiles (PCT Q1–Q4), with the exception of fasting glucose in PCT (Q1), creatinine, mean platelet volume (MPV) and PDW in PCT (Q3), and tHcy in PCT(Q4), there were no significant differences in any other baseline characteristics between the enalapril-folic acid and enalapril groups in each PCT quartile (Supplementary Table 1).

**Table 1:** Baseline characteristics of study participants by *MTHFR* C677T genotype

*MTHFR* C677T genotype				
	*MTHFR* 677 CC/CT		*MTHFR* 677 TT	
	(*N* = 8173)		(*N* = 3012)	
	Enalapril group	Enalapril–folic acid group	Enalapril group	Enalapril–folic acid group
*N*	4076	4097	1507	1505
Sex				
Male, *n* (%)	1583 (38.84%)	1546 (37.73%)	576 (38.22%)	593 (39.40%)
Age, years	59.37 ± 7.65	59.52 ± 7.51	59.64 ± 7.48	59.42 ± 7.51
Body mass index, kg/m^2^	25.58 ± 3.59	25.62 ± 3.61	25.61 ± 3.57	25.66 ± 3.58
Baseline SBP, mm Hg	168.14 ± 20.83	167.81 ± 20.61	168.35 ± 21.30	167.99 ± 21.23
Baseline DBP, mm Hg	94.98 ± 12.06	94.93 ± 11.80	95.16 ± 12.09	95.39 ± 11.28
Smoking status				
Never	2837 (69.60%)	2910 (71.03%)	1087 (72.13%)	1058 (70.30%)
Former	339 (8.32%)	292 (7.13%)	104 (6.90%)	124 (8.24%)
Current	900 (22.08%)	895 (21.85%)	316 (20.97%)	323 (21.46%)
Alcohol consumption				
Never	2882 (70.71%)	2955 (72.13%)	1093 (72.53%)	1071 (71.16%)
Former	278 (6.82%)	259 (6.32%)	98 (6.50%)	100 (6.64%)
Current	916 (22.47%)	883 (21.55%)	316 (20.97%)	334 (22.19%)
Laboratory tests				
Total cholesterol, mmol/l	5.64 ± 1.17	5.68 ± 1.19	5.66 ± 1.20	5.63 ± 1.16
Triglycerides, mmol/l	1.70 ± 0.97	1.73 ± 1.81	1.73 ± 0.97	1.68 ± 1.02
HDL-C, mmol/l	1.32 ± 0.36	1.33 ± 0.37	1.31 ± 0.36	1.32 ± 0.36
Fasting glucose^#^, mmol/l	6.06 ± 1.76	6.04 ± 1.78	6.17 ± 1.95	6.10 ± 1.86
Creatinine, umol/l	64.63 ± 16.37	64.99 ± 18.58	64.14 ± 15.24	66.20 ± 27.51**
tHcy^#^, umol/l	12.73 ± 5.20	12.87 ± 5.25	19.69 ± 13.54	19.95 ± 13.57
Folic acid^#^, ng/ml	8.16 ± 3.34	8.09 ± 3.15	7.04 ± 3.13	6.95 ± 3.08
PCT, %	0.19 ± 0.05	0.19 ± 0.06	0.19 ± 0.06	0.19 ± 0.05
MPV, fL	7.63 ± 1.44	7.67 ± 1.76	7.65 ± 1.64	7.63 ± 0.66
PDW, %	15.52 ± 0.39	15.54 ± 0.32**	15.57 ± 1.09	15.54 ± 0.32
PLT, *10^9^/l	255.77 ± 84.92	253.06 ± 69.74	256.19 ± 70.74	258.58 ± 71.65

Values are number (%) or mean ± SD

*DBP* diastolic blood pressure, *HDL-C* high-density lipoprotein cholesterol, *MPV* mean platelet volume, *PCT* plateletcrit, *PDW* platelet distribution width, *PLT* platelet count, *SBP* systolic blood pressure, *tHcy* total homocysteine, *MTHFR* methylenetetrahydrofolate reductase

**Indicates *p* value < 0.05 among the treatment groups (enalapril vs. enalapril-folic acid); ^#^indicates *p* value <0.05 among the *MTHFR* C677T genotypes (CC/CT vs. TT)

### First stroke risk in different platelet activation parameters and *MTHFR* C677T genotype subgroups

**Fig. 1:**
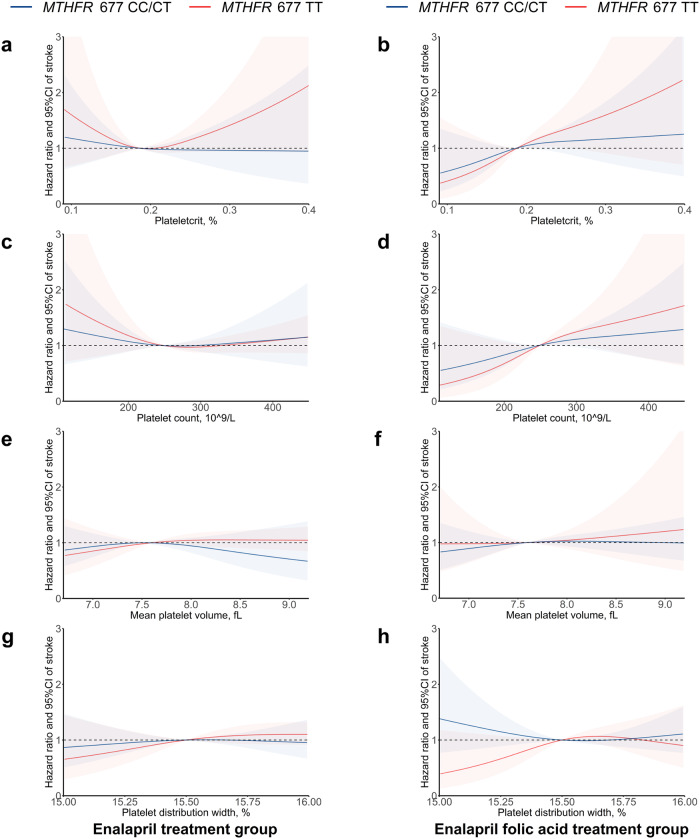
Smoothing plots of platelet activation parameters and first stroke risk by *MTHFR* C677T genotypes among treatment groups. Smoothing plots of platelet activation parameters and first stroke risk by *MTHFR* C677T genotypes among the enalapril group (**a**, **c**, **e**, **g**) and the enalapril-folic acid group (**b**, **d**, **f**, **h**). **a**, **c**, **e**, **g** Smoothing plots of plateletcrit, platelet count, mean platelet volume, platelet distribution width and first stroke risk stratified by the *MTHFR* C677T genotypes (CC/CT vs. TT) among the enalapril group. **b**, **d**, **f**, **h** Smoothing plots of plateletcrit, platelet count, mean platelet volume, platelet distribution width and first stroke risk stratified by the *MTHFR* C677T genotypes (CC/CT vs. TT) among the enalapril-folic acid group

Patients with *MTHFR* 677 CC/CT genotype who received folic acid treatment to prevent stroke occurrence had a better response. However, patients with *MTHFR* 677 TT genotype who received folic acid treatment to prevent the first stroke risk were less effective (Supplementary Fig. 2). Although elevated tHcy is an important risk factor for stroke in populations with *MTHFR* 677 CC/CT genotypes, our analyses found that tHcy level is not an useful indicator for the benefits of folic acid treatment in *MTHFR* 677 TT genotype population (Supplementary Table 2). Furthermore, we investigated the effect of platelet activation parameters on the prevention of first stroke in patients with *MTHFR* C677T genotype who received or did not receive folic acid treatment. Figure 1 and Supplementary Fig. 3 show a non-linear relationship between platelet activation parameters and first stroke and ischemic stroke risk stratified by the *MTHFR* C677T genotype among the two treatment groups. In the enalapril group, the hazard ratios of stroke and ischemic stroke were higher with lower PCT and PLT (Fig. 1 and Supplementary Fig. 3a, c). In the enalapril folic acid group, the stroke and ischemic stroke risk showed a significant reduction among participants with lower PCT and PLT, especially those with the TT genotype, suggesting that folic acid treatment is more beneficial for patients with lower PCT or PLT and the TT genotype (Fig. 1 and Supplementary Fig. 3b, d). However, there were no apparent stroke and ischemic stroke risk reduction in relation to decreasing MPV and PDW in either the enalapril group (Fig. 1 and Supplementary Fig. 3e, g) or the enalapril folic acid group (Fig. 1 and Supplementary Fig. 3f, h).

### *MTHFR* C677T genotypes and PCT on the effect of folic acid treatment in prevention of first stroke

Table 2 and Supplementary Table 3 further quantify the effect of the platelet activation parameters PCT, PLT, MPV, and PDW on folic acid treatment to reduce stroke risk based on *MTHFR* C677T genotype stratification. In patients with *MTHFR* 677 TT genotype, folic acid treatment had no significant benefit in preventing the first stroke occurrence (HR: 0.87; 95% CI: 0.61–1.24; *p* = 0.442) (Table 2). folic acid treatment among patients with the *MTHFR* 677 TT genotype showed no benefit in reducing the risk of first stroke. However, when further assessed on the basis of PCT quartile stratification, the subgroup with low PCT (Q1) and the *MTHFR* 677 TT genotype had the highest stroke and ischemic stroke incidence rate (5.6 and 5.1%). With folic acid treatment, the first stroke and ischemic stroke hazard ratio in the highest risk group [low PCT(Q1) and TT genotype] was reduced by 66% (HR: 0.34; 95% CI: 0.14–0.82; *p* = 0.016) and 67% (HR: 0.33; 95% CI: 0.13–0.83; *p* = 0.019). In the highest risk group, folic acid treatment showed significantly the best effect on decreasing the risk of first stroke (NNT = 27). Tests of interaction between PCT (Q1 vs. Q2–Q4) and *MTHFR* 677 TT genotype subgroup and folic acid treatment on first stroke (*p* = 0.021) and ischemic stroke (*p* = 0.018) were statistically significant. Furthermore, Table 2 also shows that patients with low PCT (Q1) and normal *MTHFR* 677 genotype (CC/CT) subgroups receiving folic acid reduced 60% (HR: 0.40; 95% CI: 0.23–0.70; *p* = 0.001) and 70% (HR: 0.30; 95% CI: 0.16–0.57; *p* < 0.001) stroke and ischemic stroke risk during the trial period, respectively. Tests of interaction effect between the low PCT(Q1) and the *MTHFR* 677 normal genotype (CC/CT) subgroup and folic acid treatment on first stroke (*p* = 0.023) and ischemic stroke (*p* = 0.005) were statistically significant. However, the risk reduction was not statistically significant for any of the other subgroups. Supplementary Table 3 shows that the PLT quartile was not able to screen out the *MTHFR* 677 TT genotype subgroup in which folic acid treatment significantly decreased stroke risk. In the low PLT (Q1) and normal *MTHFR* 677 genotype (CC/CT) subgroups, folic acid treatment decreased in a 59% (HR: 0.41; 95% CI: 0.23–0.72; *p* = 0.002) and 65% (HR: 0.35; 95% CI: 0.18–0.66; *p* = 0.001) risk of first stroke and ischemic stroke. In contrast, the risk reduction was not statistically significant for any of the other subgroups. Folic acid treatment in the MPV(Q2) and the *MTHFR* 677 CC/CT genotype subgroup reduced stroke risk by 43% (HR: 0.57; 95% CI: 0.36–0.91; *p* = 0.019), but the other subgroups were not significant. Folic acid treatment in the MPV(Q1–2) and the *MTHFR* 677 CC/CT genotype subgroup reduced ischemic stroke risk by 44% (HR: 0.56; 95% CI: 0.33–0.95; *p* = 0.032) and 49% (HR: 0.51; 95% CI: 0.30–0.85; *p* = 0.010), but the other subgroups were not significant. In the subgroup with PDW (Q2) and the *MTHFR* 677 TT genotype, folic acid treatment had a 2.31-fold increased risk of stroke (HR: 2.31; 95% CI: 1.06–5.04; *p* = 0.035), while folic acid treatment within the PDW (Q4) and the *MTHFR* 677 TT genotype subgroup reduced stroke risk by 59% (HR: 0.41; 95% CI: 0.20–0.85; *p* = 0.016). Folic acid treatment within the PDW (Q4) and the *MTHFR* 677 TT genotype subgroup reduced ischemic stroke risk by 53% (HR: 0.47; 95% CI: 0.23–0.98; *p* = 0.043). Nevertheless, the other subgroups were not significant. These results suggest that among platelet activation parameters, PCT is the best biomarker for identifying those hypertension patients with the TT genotype who may benefit the most from folic acid treatment.

**Table 2:** Effect of folic acid treatment on first stroke risk, stratified by *MTHFR* C677T genotypes and plateletcrit

*MTHFR* C677T genotypes subgroups	Enalapril group		Enalapril–folic acid group		NNT	Unadjusted model			Adjusted model		
TT genotype	Total	Events(%)	Total	Events(%)		HR(95% CI)	*p* value	*p* value for interaction	HR(95% CI)	*p* value	*p* value for interaction*
Total stroke											
Total	1507	66(4.4)	1505	56(3.7)	143	0.85(0.60, 1.22)	0.380		0.87(0.61, 1.24)	0.442	
PCT Q1	393	22(5.6)	363	7(1.9)	27	0.34(0.15, 0.81)	0.014	Ref	0.34(0.14, 0.82)	0.016	Ref
PCT Q2–Q4	1114	44(4.0)	1142	49(4.3)	–	1.09(0.73, 1.64)	0.669	0.011	1.09(0.72, 1.64)	0.681	0.021
PCT Q2	359	9(2.5)	371	16(4.3)	–	1.73(0.80, 3.93)	0.186		1.62(0.71, 3.72)	0.254	
PCT Q3	380	18(4.7)	404	16(4.0)	143	0.85(0.43, 1.67)	0.634		0.866(0.44,1.72)	0.678	
PCT Q4	375	17(4.5)	367	17(4.6)	–	1.01(0.52, 1.99)	0.967		0.90(0.45, 1.80)	0.763	
Ischemic stroke											
Total	1507	56(3.7)	1505	51(3.4)	333	0.92(0.63, 1.34)	0.650		0.94(0.65, 1.38)	0.767	
PCT Q1	393	20(5.1)	363	6(1.7)	29	0.32(0.13, 0.81)	0.016	Ref	0.33(0.13, 0.83)	0.019	Ref
PCT Q2–Q4	1114	36(3.2)	1142	45(3.9)	–	1.23(0.79, 1.90)	0.359	0.010	1.23(0.79, 1.91)	0.353	0.018
PCT Q2	359	8(2.2)	371	13(3.5)	–	1.58(0.65, 3.81)	0.310		1.62(0.66, 3.97)	0.292	
PCT Q3	380	12(3.2)	404	15(3.7)	–	1.20(0.56, 2.56)	0.643		1.23(0.57, 2.66)	0.606	
PCT Q4	375	16(4.3)	367	17(4.6)	–	1.08(0.55, 2.14)	0.827		0.95(0.47, 1.93)	0.888	
CC&CT genotype											
Total stroke											
Total	4076	153(3.8)	4097	110(2.7)	91	0.71(0.56, 0.91)	0.006		0.71(0.56, 0.91)	0.006	
PCT Q1	1084	45(4.2)	1044	18(1.7)	40	0.41(0.24,0.70)	0.001	Ref	0.40(0.23, 0.70)	0.001	Ref
PCT Q2–Q4	2992	108(3.6)	3053	92(3.0)	167	0.83(0.63, 1.10)	0.201	0.021	0.83(0.63, 1.10)	0.194	0.023
PCT Q2	984	30(3.0)	997	27(2.7)	333	0.89(0.53, 1.49)	0.649		0.92(0.55, 1.56)	0.759	
PCT Q3	1003	37(3.7)	1016	33(3.2)	200	0.88(0.55, 1.41)	0.604		0.84(0.52, 1.35)	0.467	
PCT Q4	1005	41(4.1)	1040	32(3.1)	100	0.75(0.47, 1.19)	0.226		0.69(0.43, 1.10)	0.117	
Ischemic stroke											
Total	4076	134(3.3)	4097	92(2.2)	90	0.68(0.52, 0.89)	0.004		0.67(0.52, 0.88)	0.004	
PCT Q1	1084	41(3.8)	1044	12(1.1)	37	0.30(0.16, 0.56)	<0.001	Ref	0.30(0.16, 0.57)	<0.001	Ref
PCT Q2–Q4	2992	93(3.1)	3053	80(2.6)	200	0.84(0.63, 1.14)	0.262	0.004	0.83(0.62, 1.13)	0.235	0.005
PCT Q2	984	24(2.4)	997	25(2.5)	–	1.03(0.59, 1.8)	0.923		1.07(0.61, 1.88)	0.818	
PCT Q3	1003	34(3.4)	1016	29(2.9)	200	0.84(0.52, 1.39)	0.504		0.80(0.49, 1.32)	0.384	
PCT Q4	1005	35(3.5)	1040	26(2.5)	100	0.72(0.43, 1.19)	0.195		0.64(0.38, 1.07)	0.088	

PCT Q1 (≤0.16%), Q2–Q4 (>0.16%), PCT Q2 (>0.16%–≤0.186%), PCT Q3 (>0.186%–≤0.217%), PCT Q4 (>0.217%). Variables in the adjusted model include age, sex, smoking status, body mass index, systolic blood pressure, diastolic blood pressure, total cholesterol, triglycerides, high-density lipoprotein cholesterol, total homocysteine, creatinine, and blood glucose. Enalapril group is the reference group

*CI* confidence interval, *HR* hazard ratio, *MTHFR* methylenetetrahydrofolate reductase, *NNT* number needed to treat, *PCT* plateletcrit, *Q* quartile

*p value for interaction test: two-way interaction of PCT (Q1 vs. Q2–Q4) and treatment groups (enalapril vs. enalapril-folic acid) on first stroke

## Discussion

**Fig. 2:**
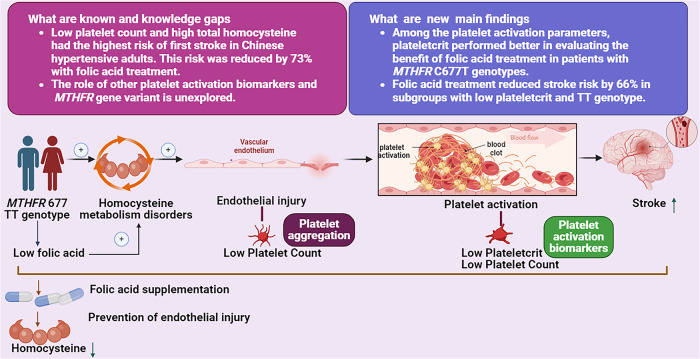
Effects of plateletcrit and the *MTHFR* 677 TT genotype on folic acid treatment for first stroke prevention. Among the *MTHFR* 677 TT genotype population, total homocysteine metabolism dysfunction causes vascular endothelial injury, accelerates platelet aggregation and activation, thus leading to a large consumption of platelets and a decrease in plateletcrit, which ultimately promotes thrombosis and stroke. Folic acid treatment can prevent endothelial damage, reduce total homocysteine levels and the first stroke risk in the TT genotype populations. This figure was created with the aid of Biorender (https://biorender.com/)

In this study, for the first time, we evaluated the protective effect of folic acid treatment on stroke by simultaneous consideration of *MTHFR* C677T genotypes and all platelet activation parameters in hypertensive patients, which has never been reported before. This study revealed two main findings. First, we evaluated the platelet activation parameters PCT, PLT, MPV, and PDW as indicative function of folic acid treatment among the *MTHFR* C677T genotypes patients. Our analysis showed that PCT may be better for evaluating the effect of folic acid treatment on reducing the risk of first stroke in hypertensive patients with *MTHFR* C677T genotype, especially those with the TT genotype. Second, we found that among those without folic acid treatment, patients with both low PCT (Q1) and *MTHFR* 677 TT genotype had the highest stroke incidence rate (5.6%). Low PCT (Q1) and *MTHFR* 677 TT genotype highest risk subgroup received folic acid treatment which benefited the most. Low PCT (Q1) can also be used to screen the benefit of folic acid treatment to reduce the first stroke risk in patients with *MTHFR* 677 CC/CT genotypes. Taken together, PCT can be regarded as a significant biomarker to identify those who would greatly benefit from folic acid treatment, especially for *MTHFR* 677 TT genotype patients, a subgroup known to have the highest stroke risk (Fig. 2).

The *MTHFR* 677 TT genotype frequency in the Chinese populations is higher than that in other populations; the other ethnicities populations’ *MTHFR* 677 TT genotype frequency is 10 to 12%, but it is as high as 25% in the Chinese hypertensive population.^25,26^ As an important rate-limiting enzyme in tHcy and folate metabolism, individuals carrying the *MTHFR* 677 TT genotype have a 70% reduction in activity, which decreases the ability of tHcy to remethylate to methionine, resulting in a disturbance of the folic acid metabolic cycle and folic acid deficiency, as well as hyperhomocysteinemia.^7^ These metabolic and physiological changes can greatly increase stroke occurrence. In this study, we found that patients with the *MTHFR* 677 TT genotype had a higher risk of first stroke compared to patients with the *MTHFR* 677 CC/CT genotype. In addition, folic acid utilization efficiency was decreased in the *MTHFR* 677 TT genotype patients. Therefore, it is particularly important to find an effective test for hypertension patients with *MTHFR* 677 TT genotype to identify those who benefit from folic acid in order to better prevent stroke.

Hyperhomocysteinemia is deemed as a crucial risk factor for stroke. The 2021 American Guidelines for the Secondary Prevention of Stroke state that folic acid treatment can decrease the incidence of stroke.^13^ While folic acid contribute to decrease tHcy, its effectiveness, however, may differ depending on the *MTHFR* C677T genotype. Huo et al.^2^ showed that even though tHcy levels in TT genotype hypertensive patients were significantly higher than those in CC/CT genotype hypertensive patients. Although elevated tHcy is an important risk factor for stroke in populations with CC/CT genotypes. Our analysis found that tHcy level did not contribute to additional information in screening the benefits of folic acid treatment in *MTHFR* 677 TT genotype population. None of the quartiles of tHcy levels showed any response to folic acid treatment within the TT genotype group. The use of simple and universal indicators and methods in clinical treatment to find these more beneficial groups will help popularize precision targeted therapy efficiently. At the same time, it also provides useful enlightenment for deepening the targeted therapy mechanism and biological path scientific exploration.

As the most common cerebrovascular disease, the pathological process of stroke is closely related to thrombosis and platelet activation. Studies have reported that population with activated platelets have a higher risk of stroke, as platelet activation is closely related to the stroke pathophysiological mechanism.^27–29^ Endothelial dysfunction and platelet activation are important mediators of thrombosis in atherosclerosis. Hypertension give rise to vascular endothelial injury through shear stress, triggering coagulation and fibrinolysis systems.^30^ Activated platelets recruited monocytes to the walls of blood vessels and then evolved into macrophages, promoting the atherosclerosis.^31^ When active atherosclerotic plaques rupture, circulating platelets are exposed to subcutaneous collagen, fibronectin, and von Willebrand factor, stimulating platelet activation and eventually thrombosis.^32^ Platelet activation is also affected by tHcy. In the case of hyperhomocysteinemia, increased hydrogen sulfide in platelets triggered the arachidonic acid cascade pathway, promoting increased TXA2 production and leading to changes in platelet volume and capacity.^33^ Elevated tHcy is associated with increased platelet activity, manifested by increased secretion of soluble CD40L and β-thromboglobulin after microvascular injury.^14^ tHcy promotes thrombosis by activating platelets and the clotting pathway.^34^ Therefore, the process of platelet activation and thrombosis may partially mediate the effect of hyperhomocysteinemia on stroke progression.

Folic acid can inhibit ERK1/2/NOX4/ROS pathway to reduce peroxide production, enhance antioxidant enzyme activity to attenuate oxidative stress and inflammation, and thus play a protective role on stroke.^35^ More importantly, folic acid may alleviate thromboxane A2 release and clotting factor expression by decreasing tHcy, thereby reducing platelet activation and thrombosis, and ultimately preventing stroke. Undas et al.^14^ had found that elevation of tHcy may accelerate platelet activation induced by vascular injury, while folic acid treatment can eliminate platelet hyperreactivity.^14^ Previous studies have also reported that combining PLT with serum tHcy levels can significantly improve the indicator power of the folic acid on stroke prevention.^17^ Based on this evidence, we speculate that platelet activation parameters such as PCT may indirectly reflect endothelial injury and the benefit of folic acid treatment. Hypertensive patients with low PCT may already have endothelial cell damage, resulting in reduced platelet consumption, so this group of population at higher risk of stroke. Folic acid treatment is particularly important.

In this study, we found that PCT was superior to other platelet activation parameters for assessing those who may benefit most from folic acid treatment, especially for patients with the *MTHFR* 677 TT genotype who are at the highest stroke risk. This finding has the potential to guide the clinical management of patients with the *MTHFR* C677T genotype, but further prospective studies will be needed to confirm this finding.

This study has the following strengths: first, our study used the largest data from the folic acid intervention in stroke primary prevention trial (CSPPT). This study is strengthened by its large sample size, derived from a high-quality randomized folic acid clinical trial that for the first time incorporated *MTHFR* C677T genotypes in the randomization.^2^ We comprehensively analyzed the prospective association between platelet activation parameters (PCT, PLT, MPV, and PDW) and the first stroke risk in patients with *MTHFR* C677T genotype, and determined that PCT assessment was the most useful. Second, the study population consisted of Chinese adults with hypertension, with a higher prevalence of TT genotypes compared to Western populations (25% in China compared to 10 to 12% in America).^7,9^ Third, our study is the first to evaluate folic acid benefit situation on stroke prevention by performing a comprehensive analysis of platelet activation parameters and the *MTHFR* C677T genotype. Our finding that patients in the subgroup of low PCT and with the TT genotype benefited the most from folic acid treatment. Despite its many advantages, there are still some limitations in this study. First, our current study only analyzed baseline levels of platelet activation parameters. The dynamic changes of platelet activation parameters in patients during follow-up will also need to be monitored in the future. Second, 0.8 mg/day of folic acid was selected for this study, therefore, we were unable to assess whether higher doses of folic acid would benefit more patients with different *MTHFR* C677T genotypes and tHcy levels. In the end, this is a post hoc study, our study findings might not be generalizable to whole China and could be perceived as hypotheses-generating, further extensive research would be needed.

## Conclusions

In this study, we found that low PCT can further help identify who would greatly benefit from folic acid treatment to reduce the first stroke risk, particularly in hypertensive patients with the TT genotype who are at the highest risk of stroke. Therefore, PCT has the potential to be a biomarker for evaluating folic acid efficacy in hypertensive patients with the *MTHFR* 677 TT genotype.

## Materials and methods

All participants in this study were from the China Stroke Primary Prevention Trial (CSPPT) (NCT00794885). Our paper follows the practice of the Journal of the American Heart Association in implementing transparency and openness promotion guidelines. The CSPPT was approved by the Ethics Committee of the Institute of Biomedical Research of Anhui Medical University in Hefei, China. (FWA assurance number FWA00001263). All participants signed a written, informed consent.

In short, CSPPT was a multi-community, randomized, double-blind, controlled trial. The study was conducted at 32 community research centers in Jiangsu and Anhui provinces of China from May 19, 2008 to August 24, 2013. Eligible participants’ inclusion criteria included: (1) Age 45–75 years old; (2) Sitting blood pressure criteria were met at both screening visits and enrollment visits (at least 24 h apart) : seated, resting systolic blood pressure ≥140 mmHg, or diastolic blood pressure ≥90 mmHg, or currently receiving antihypertensive medications. The main exclusion criteria included: (1) Previous history of stroke; (2) History of myocardial infarction; (3) Patients with confirmed secondary hypertension; (4) suffering from congenital or acquired organic heart disease; (5) Previous history of heart failure; (6) suffering from serious systemic physical diseases, unable to cooperate with the completion of the interview.

Eligible participants were first stratified by the *MTHFR* C677T genotype (CC, CT, or TT), and then were randomly assigned in a 1:1 ratio within each genotype group to receive one of two oral treatments daily: one tablet of 10 mg enalapril and 0.8 mg folic acid, or one tablet of 10 mg enalapril. During the trial, concurrent use of other antihypertensive drugs (mainly calcium channel blockers or diuretics) was allowed, but B vitamins were not allowed. Participants were scheduled for follow-up every 3 months.

### Laboratory evaluation

Venous blood samples were collected from all participants after fasting for more than 10 h. Laboratory testing was conducted at the National Clinical Research Center for Kidney Diseases laboratory (Nanfang Hospital, Guangzhou, China).^2^ BC-3200 hematology analyzer (Mindray Medical, Shenzhen, China) measured a complete baseline blood cell count, including PCT, PLT, mean platelet volume (MPV) and platelet distribution width (PDW). Fasting blood glucose, total cholesterol, high-density lipoprotein cholesterol [HDL-C], triglycerides, tHcy, creatinine and *MTHFR* C677T genotypes were obtained by a fully automated clinical analyzer (Beckman Coulter, Brea, California) and an ABI Prism 7900HT sequence detection system (Life Technologies, Carlsbad, California). Serum folic acid was measured through using a chemiluminescent immunoassay in the commercial laboratory (New Industry, Shenzhen, China).

### Outcomes

The primary outcome event of this study was symptomatic stroke (including ischemic stroke, hemorrhagic stroke and stroke with uncertain subtype, but excluding subarachnoid hemorrhage and silent stroke) that occurred for the first time during entry into randomized treatment.^2^ The experts of the outcome event adjudication board judge the occurrence of the outcome event.^2^

### Covariables

The adjusted model in this study mainly adjusts the following variables: age, sex, smoking status, body mass index, baseline systolic and diastolic blood pressure, total cholesterol, triglycerides, and HDL-C, tHcy, glucose, creatinine. These covariates were selected on the basis of the original CSPPT study results, and details on the definitions of each covariable have been previously reported.^2^

### Statistical analysis

According to *MTHFR* C677T genotype grouping, continuous variables and categorical variables data in this study, we used mean ± SD and frequency (%) to represent, respectively. Kaplan–Meier curves (log-rank test) were used to evaluate the benefit of first stroke in *MTHFR* C677T genotype patients receiving folic acid treatment. We used a Cox proportional hazard model of unadjusted and adjusted for correlated variables to estimate the hazard ratio and 95% confidence interval for stroke occurrence in folic acid treatment subgroups defined by the *MTHFR* C677T genotype combined with tHcy levels or platelet activation parameters, and tested their interactions. A receiver operating curve of platelet activation parameters and stroke risk was plotted for those in the enalapril folic acid group, as well as a restricted cubic spline curve based on a multifactor Cox regression model. *p* < 0.05 was considered statistically significant. In this study, R version 4.1.2 (R Foundation for Statistical Computing, Vienna, Austria) and Empower (X&Y Solutions, Inc. Boston, Massachusetts) were used for data analysis.

### Supplementary information

Supplementary_Materials-2024.3.1-pct-manuscript.docx

## Data Availability

All data supporting this paper are presented in the text and Supplementary Materials. The original data sets are also available from the corresponding author upon reasonable request.
