# Supplementary material for: Effect of plateletcrit and methylenetetrahydrofolate reductase (MTHFR) C677T genotypes on folic acid efficacy in stroke prevention
Source: Signal Transduct Target Ther. 2024 May 10;9:110. doi: 10.1038/s41392-024-01817-0 (PMC11082186; doi:10.1038/s41392-024-01817-0)
Supplement: Supplementary file 1 — Supplementary_Materials-2024.3.1-pct-manuscript.docx [file 41392_2024_1817_MOESM1_ESM.docx]

Supplementary Materials for

**Effect of** **plateletcrit and** ***methylenetetrahydrofolate reductase (MTHFR)* C677T genotypes on folic acid efficacy in stroke prevention**

Yuncong Shi, MD,^a#^ Zhengzhipeng Zhang, MD,^a#^ Binyan Wang, PhD,^b^ Yu Wang, PhD,^c^ Xiangyi Kong, MD,^d^ Yong Sun, PhD,^e^ Aimin Li, PhD,^e^ Yimin Cui, PhD,^f^ Yan Zhang, PhD,^g^ Jianping Li, PhD,^g^ Yong Huo, PhD,^g^ Hui Huang, PhD,^a*^

# joint author

Correspondence to: huangh8@mail.sysu.edu.cn.

**This PDF file includes:**

Figures. S1 to S3

Tables S1 to S3

Received Enalapril alone
(n=4076)

Received Enalapril-Folic Acid
(n=4097)

Received Enalapril alone
(n=1507)

Received Enalapril-Folic Acid
(n=1505)

*MTHFR* 677 CC/CT genotype

(n=8173)

*MTHFR* 677 TT genotype

(n=3012)

Participants randomized in the CSPPT trial (n=20,702)

Participants from Anqing center, due to no baseline plateletcrit data(n=5,216)

Participants included in final analysis (n=11185)

Participants from Lianyungang center (n=15,486)

Participants excluded due to missing baseline platelet parameters or total homocysteine data or with antiplatelet drug use (n=4301)

**Figure. S1 Flow Chart of the Study Participants** A flow chart of the study participants is shown, including the number screened, excluded, groupings, and the number who completed the randomized, controlled clinical trial.


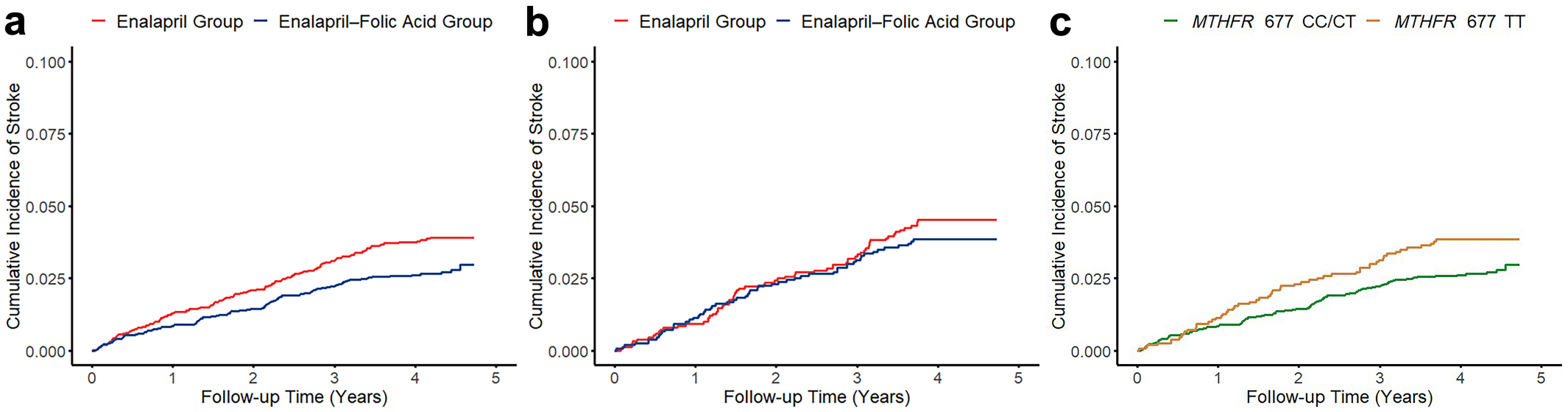


**Figure. S2. Kaplan-Meier curves of first stroke cumulative incidence stratified by *MTHFR* C677T genotypes and treatment group**

Kaplan-Meier curves of cumulative incidence of first stroke stratified by *MTHFR* C677T genotypes (CC/CT vs TT) and treatment groups (Enalapril vs. Enalapril-Folic Acid). (a) Kaplan-Meier curves of cumulative incidence of first stroke stratified by the *MTHFR* 677 CC/CT genotypes and treatment groups (Enalapril vs. Enalapril-Folic Acid); (b) Kaplan-Meier curves of cumulative incidence of first stroke stratified by the *MTHFR* 677 TT genotype and treatment groups (Enalapril vs. Enalapril-Folic Acid); (c) Kaplan-Meier curves of cumulative incidence of first stroke stratified by the *MTHFR* C677T genotypes (CC/CT vs TT) in the Enalapril-Folic Acid group.





**Figure. S3. Smoothing plots of platelet activation parameters and first** **ischemic stroke risk by *MTHFR* C677T genotypes among the enalapril group and the enalapril-folic group**

Smoothing plots of platelet activation parameters and first ischemic stroke risk by *MTHFR* C677T genotypes among the enalapril group (panels a, c, e, g) and the enalapril-folic acid group (panels b, d, f, h). (panels a, c, e, g) Smoothing plots of plateletcrit, platelet count, mean platelet volume, platelet distribution width and first ischemic stroke risk stratified by the *MTHFR* C677T genotypes (CC/CT vs TT) among the enalapril group. (panels b, d, f, h) Smoothing plots of plateletcrit, platelet count, mean platelet volume, platelet distribution width and first ischemic stroke risk stratified by the *MTHFR* C677T genotypes (CC/CT vs TT) among the enalapril-folic acid group.

**Table S1. Baseline Characteristics of Study Participants by Plateletcrit Quartiles (N=11185)**

|  | Plateletcrit Quartiles | | | | | | | |
| --- | --- | --- | --- | --- | --- | --- | --- | --- |
|  | Q1 | | Q2 | | Q3 | | Q4 | |
|  | 2884 | | 2711 | | 2803 | | 2787 | |
|  | ≤0.16% | | ＞0.16-≤0.186% | | ＞0.186-≤0.217% | | ＞0.217% | |
|  | Enalapril Group | Enalapril–Folic Acid Group | Enalapril Group | Enalapril–Folic Acid Group | Enalapril Group | Enalapril–Folic Acid Group | Enalapril Group | Enalapril–Folic Acid Group |
| N | 1477 | 1407 | 1343 | 1368 | 1383 | 1420 | 1380 | 1407 |
| Male, n (%) | 748 (50.64%) | 690 (49.04%) | 583 (43.41%) | 571 (41.74%) | 488 (35.29%) | 515 (36.27%) | 340 (24.64%) | 363 (25.80%) |
| Age, yrs | 60.07 ± 7.47 | 60.05 ± 7.48 | 59.38 ± 7.68 | 59.43 ± 7.26 | 59.17±7.69 | 59.28 ± 7.55 | 59.10 ± 7.56 | 59.21 ± 7.72 |
| Body mass index, kg/m^2^ | 25.30 ± 3.50 | 25.23 ± 3.38 | 25.59 ± 3.50 | 25.61 ± 3.59 | 25.74±3.65 | 25.80 ± 3.69 | 25.74 ± 3.67 | 25.88 ± 3.70 |
| *MTHFR* C677T genotypes, n (%) |  |  |  |  |  |  |  |  |
| CC/CT | 1084 (73.39%) | 1044 (74.20%) | 984 (73.27%) | 997 (72.88%) | 1003 (72.52%) | 1016 (71.55%) | 1005 (72.83%) | 1040 (73.92%) |
| TT | 393 (26.61%) | 363 (25.80%) | 359 (26.73%) | 371 (27.12%) | 380 (27.48%) | 404 (28.45%) | 375 (27.17%) | 367 (26.08%) |
| Baseline SBP, mm Hg | 167.87 ± 21.29 | 167.27 ± 21.19 | 168.11±20.70 | 167.49 ± 20.28 | 168.07±21.23 | 167.94± 20.75 | 168.76±20.58 | 168.72 ± 20.85 |
| Baseline DBP, mm Hg | 94.63 ± 12.01 | 94.39 ± 12.20 | 95.68 ± 12.70 | 95.56 ± 11.53 | 95.56±11.96 | 95.30 ± 11.66 | 94.29± 11.55 | 94.99 ± 11.21 |
| Smoking status |  |  |  |  |  |  |  |  |
| Never | 933 (63.17%) | 923 (65.60%) | 906 (67.46%) | 938 (68.57%) | 988 (71.44%) | 1006 (70.85%) | 1097 (79.49%) | 1101 (78.25%) |
| Former | 164 (11.10%) | 131 (9.31%) | 115 (8.56%) | 111 (8.11%) | 91 (6.58%) | 100 (7.04%) | 73 (5.29%) | 74 (5.26%) |
| Current | 380 (25.73%) | 353 (25.09%) | 322 (23.98%) | 319 (23.32%) | 304 (21.98%) | 314 (22.11%) | 210 (15.22%) | 232 (16.49%) |
| Alcohol consumption |  |  |  |  |  |  |  |  |
| Never | 950 (64.32%) | 946 (67.24%) | 928 (69.10%) | 942 (68.86%) | 997 (72.09%) | 1019 (71.76%) | 1100 (79.71%) | 1119 (79.53%) |
| Former | 120 (8.12%) | 108 (7.68%) | 97 (7.22%) | 96 (7.02%) | 96 (6.94%) | 91 (6.41%) | 63 (4.57%) | 64 (4.55%) |
| Current | 407 (27.56%) | 353 (25.09%) | 318 (23.68%) | 330 (24.12%) | 290 (20.97%) | 310 (21.83%) | 217 (15.72%) | 224 (15.92%) |
| Laboratory results |  |  |  |  |  |  |  |  |
| Total cholesterol, mmol/L | 5.42 ± 1.11 | 5.40 ± 1.09 | 5.61 ± 1.12 | 5.66 ± 1.22 | 5.76 ± 1.21 | 5.79 ± 1.18 | 5.81 ± 1.23 | 5.82 ± 1.18 |
| Triglycerides, mmol/L | 1.62 ± 0.90 | 1.60 ± 0.95 | 1.65 ± 0.91 | 1.70 ± 1.11 | 1.78 ± 1.02 | 1.81 ± 2.72 | 1.78 ± 1.01 | 1.77 ± 1.00 |
| HDL-C, mmol/L | 1.30 ± 0.36 | 1.32 ± 0.37 | 1.34 ± 0.37 | 1.34 ± 0.38 | 1.32 ± 0.36 | 1.33 ± 0.37 | 1.32 ± 0.35 | 1.33 ± 0.36 |
| Fasting glucose, mmol/L | 6.13 ± 1.81 | 5.98 ± 1.62* | 6.03 ± 1.68 | 6.04 ± 1.84 | 6.06 ± 1.74 | 6.07 ± 1.67 | 6.13 ± 1.99 | 6.14 ± 2.05 |
| Creatinine, umol/L | 67.52 ± 18.54 | 68.01 ± 21.67 | 65.22 ± 15.08 | 65.89 ± 17.82 | 63.77 ± 14.45 | 65.51 ± 28.31* | 61.29 ± 15.05 | 61.85 ± 14.46 |
| tHcy, umol/L | 15.06 ± 9.13 | 15.07 ± 9.29 | 14.74 ± 9.41 | 15.07 ± 9.61 | 14.79 ± 9.04 | 14.52 ± 7.61 | 13.82 ± 7.77 | 14.45 ± 9.02* |
| Folic acid, ng/mL | 7.79 ± 3.19 | 7.76 ± 3.09 | 7.94 ± 3.41 | 7.82 ± 3.29 | 7.75 ± 3.17 | 7.74 ± 3.11 | 7.95 ± 3.52 | 7.80 ± 3.20 |
| MPV,fL | 7.62 ± 0.71 | 7.66 ± 1.91 | 7.63 ± 1.72 | 7.58 ± 0.64 | 7.54 ± 0.65 | 7.68 ± 2.22* | 7.74 ± 2.28 | 7.71 ± 0.66 |
| PDW, % | 15.63 ± 0.33 | 15.63 ± 0.33 | 15.54 ± 1.20 | 15.53 ± 0.29 | 15.46 ± 0.27 | 15.49 ± 0.28* | 15.51 ± 0.37 | 15.53 ± 0.36 |

Values are number (%) or mean±SD. *indicates P value＜0.05 among the treatment groups (enalapril vs. enalapril-folic acid).

DBP, diastolic blood pressure; HDL-C, high-density lipoprotein cholesterol; MPV, mean platelet volume; *MTHFR,* methylenetetrahydrofolate reductase; PDW, platelet distribution width; SBP, systolic blood pressure; tHcy, total homocysteine.

**Table S2. Effect of *MTHFR* C677T genotypes and total homocysteine on folic acid treatment in prevention of first stroke**

|  | Enalapril Group | | Enalapril–Folic Acid Group | | NNT | Unadjusted Model | | | Adjusted Model | | |
| --- | --- | --- | --- | --- | --- | --- | --- | --- | --- | --- | --- |
| *MTHFR* C677T genotype Subgroups | Total | Events(%) | Total | Events(%) |  | HR(95%CI) | p value | p value for interaction | HR(95%CI) | p value | p value for interaction^*^ |
| CC &CT |  |  |  |  |  |  |  | 0.146 |  |  | 0.094 |
| Total | 4076 | 153(3.8) | 4097 | 110(2.7) | 91 | 0.710(0.556, 0.907) | 0.006 |  | 0.710(0.555, 0.908) | 0.006 |  |
| tHcy Q1(≤10.320) | 1223 | 33(2.7) | 1201 | 23(1.9) | 125 | 0.707(0.415, 1.203) | 0.201 |  | 0.783(0.458, 1.338) | 0.371 |  |
| tHcy Q2(＞10.320-≤12.480) | 1159 | 30(2.6) | 1113 | 29(2.6) | - | 1.005(0.603, 1.674) | 0.985 |  | 1.031(0.616, 1.728) | 0.907 |  |
| tHcy Q3(＞12.480-≤15.700) | 1067 | 51(4.8) | 1062 | 38(3.6) | 83 | 0.745(0.489, 1.134) | 0.169 |  | 0.765(0.500, 1.171) | 0.218 |  |
| tHcy Q4(＞15.700) | 627 | 39(6.2) | 721 | 20(2.8) | 29 | 0.435(0.254, 0.745) | 0.002 |  | 0.442(0.257, 0.759) | 0.003 |  |
| TT |  |  |  |  |  |  |  | 0.272 |  |  | 0.241 |
| Total | 1507 | 66(4.4) | 1505 | 56(3.7) | 143 | 0.852(0.597, 1.217) | 0.380 |  | 0.867(0.606,1.240) | 0.434 |  |
| tHcy Q1(≤10.320) | 198 | 8(4.0) | 188 | 10(5.3) | - | 1.317(0.520, 3.336) | 0.562 |  | 1.257(0.469,3.368) | 0.649 |  |
| tHcy Q2(＞10.320-≤12.480) | 250 | 13(5.2) | 265 | 7(2.6) | 38 | 0.508(0.203, 1.274) | 0.149 |  | 0.522(0.204,1.333) | 0.174 |  |
| tHcy Q3(＞12.480-≤15.700) | 331 | 16(4.8) | 334 | 9(2.7) | 48 | 0.553(0.244, 1.251) | 0.155 |  | 0.508(0.221,1.169) | 0.111 |  |
| tHcy Q4(＞15.700) | 728 | 29(4.0) | 718 | 30(4.2) | - | 1.059(0.636, 1.764) | 0.826 |  | 1.095(0.656,1.829) | 0.728 |  |

Adjusted model adjusted for: age, sex, smoking status, body mass index, systolic blood pressure, diastolic blood pressure, total cholesterol, triglycerides, high-density lipoprotein cholesterol, creatinine, and blood glucose. Enalapril group is the reference group. *p value for interaction test: 2-way interaction of tHcy (Q1-Q4) and treatment groups (enalapril vs. enalapril-folic acid) on first stroke.

CI, confience interval; HR, hazard ratio; NNT, number need to treat; tHcy, total homocysteine; Q, quartile.

**Table S3. Effect of platelet activation parameters and *MTHFR* C677T genotype on folic acid treatment in the prevention of first stroke**

|  | Enalapril Group | | Enalapril–Folic Acid Group | | NNT | Unadjusted Model | | | Adjusted Model | | |
| --- | --- | --- | --- | --- | --- | --- | --- | --- | --- | --- | --- |
| *MTHFR* C677T Genotype Subgroups | Total | Events(%) | Total | Events(%) |  | HR(95%CI) | p value | p value for interaction | HR(95%CI) | p value | p value for interaction^*^ |
| CC&CT |  |  |  |  |  |  |  |  |  |  |  |
| Total stroke |  |  |  |  |  |  |  |  |  |  |  |
| PLT Q1(≤210*10^9^/L) | 1021 | 42 (4.1%) | 973 | 17 (1.7%) | 42 | 0.41(0.24, 0.73) | 0.002 | 0.128 | 0.41(0.23, 0.72) | 0.002 | 0.174 |
| PLT Q2(＞210-≤248*10^9^/L) | 968 | 33 (3.4%) | 1011 | 31 (3.1%) | 333 | 0.90(0.55, 1.47) | 0.664 |  | 0.94(0.57, 1.54) | 0.803 |  |
| PLT Q3(＞248-≤291*10^9^/L) | 962 | 32 (3.3%) | 1005 | 26 (2.6%) | 143 | 0.78(0.46, 1.31) | 0.339 |  | 0.72 (0.43, 1.23) | 0.228 |  |
| PLT Q4(＞291*10^9^/L) | 971 | 41 (4.2%) | 956 | 33 (3.5%) | 143 | 0.82(0.52, 1.30) | 0.395 |  | 0.78 (0.49, 1.23) | 0.283 |  |
| Ischemic stroke |  |  |  |  |  |  |  |  |  |  |  |
| PLT Q1(≤210*10^9^/L) | 1021 | 37(3.6%) | 973 | 13(1.3%) | 43 | 0.36(0.19, 0.68) | 0.001 | 0.152 | 0.35(0.18, 0.66) | 0.001 | 0.223 |
| PLT Q2(＞210-≤248*10^9^/L) | 968 | 28(2.9%) | 1011 | 28(2.8%) | 1000 | 0.96(0.57, 1.62) | 0.869 |  | 1.01(0.60, 1.71) | 0.972 |  |
| PLT Q3(＞248-≤291*10^9^/L) | 962 | 29(3.0%) | 1005 | 22(2.2%) | 125 | 0.73(0.42, 1.26) | 0.256 |  | 0.67(0.38, 1.17) | 0.160 |  |
| PLT Q4(＞291*10^9^/L) | 971 | 35(3.6%) | 956 | 27(2.8%) | 125 | 0.79(0.48, 1.30) | 0.344 |  | 0.73(0.44,1.21) | 0.217 |  |
| TT |  |  |  |  |  |  |  |  |  |  |  |
| Total stroke |  |  |  |  |  |  |  |  |  |  |  |
| PLT Q1(≤210*10^9^/L) | 378 | 20 (5.3%) | 352 | 9 (2.6%) | 37 | 0.48(0.22, 1.06) | 0.087 | 0.083 | 0.46 (0.21, 1.04) | 0.06 | 0.112 |
| PLT Q2(＞210-≤248*10^9^/L) | 348 | 12 (3.4%) | 372 | 11 (3.0%) | 250 | 0.86(0.38, 1.95) | 0.719 |  | 0.79 (0.34, 1.83) | 0.585 |  |
| PLT Q3(＞248-≤291*10^9^/L) | 362 | 16 (4.4%) | 373 | 18 (4.8%) | - | 1.11(0.56, 2.17) | 0.770 |  | 1.12 (0.56, 2.21) | 0.751 |  |
| PLT Q4(＞291*10^9^/L) | 364 | 14 (3.8%) | 357 | 16 (4.5%) | - | 1.17(0.57, 2.39) | 0.672 |  | 1.10 (0.52, 2.31) | 0.805 |  |
| Ischemic stroke |  |  |  |  |  |  |  |  |  |  |  |
| PLT Q1(≤210*10^9^/L) | 378 | 18(4.8%) | 352 | 9(2.6%) | 45 | 0.34(0.24, 1.20) | 0.129 | 0.044 | 0.49(0.21, 1.11) | 0.088 | 0.065 |
| PLT Q2(＞210-≤248*10^9^/L) | 348 | 11(3.2%) | 372 | 10(2.7%) | 200 | 0.85(0.36, 2.01) | 0.717 |  | 0.90(0.38, 2.16) | 0.816 |  |
| PLT Q3(＞248-≤291*10^9^/L) | 362 | 12(3.3%) | 373 | 16(4.3%) | - | 1.31(0.62, 2.77) | 0.478 |  | 1.36(0.64, 2.90) | 0.431 |  |
| PLT Q4(＞291*10^9^/L) | 364 | 11(3.0%) | 357 | 16(4.5%) | - | 1.49(0.69, 3.22) | 0.306 |  | 1.45(0.65, 3.22) | 0.363 |  |
| *MTHFR* C677T Genotype Subgroups |  |  |  |  |  |  |  |  |  |  |  |
| CC&CT |  |  |  |  |  |  |  |  |  |  |  |
| Total stroke |  |  |  |  |  |  |  |  |  |  |  |
| MPV Q1 (≤7.20fL) | 1249 | 45(3.6) | 1218 | 27(2.2) | 71 | 0.61(0.38,0.99) | 0.044 | 0.354 | 0.63(0.39,1.02) | 0.061 | 0.294 |
| MPV Q2 (＞7.20-≤7.60fL) | 1067 | 51(4.8) | 973 | 27(2.8) | 50 | 0.57(0.36,0.91) | 0.019 |  | 0.57(0.36, 0.91) | 0.019 |  |
| MPV Q3(＞7.60fL-≤8.0fL) | 784 | 21(2.7) | 888 | 30(3.4) | - | 1.26(0.72,2.21) | 0.411 |  | 1.18(0.67, 2.07) | 0.566 |  |
| MPV Q4 (＞8.0fL) | 976 | 36(3.7) | 1018 | 26(2.6) | 91 | 0.69(0.41, 1.13) | 0.141 |  | 0.73(0.44, 1.23) | 0.238 |  |
| Ischemic stroke |  |  |  |  |  |  |  |  |  |  |  |
| MPV Q1 (≤7.20fL) | 1249 | 39(3.1) | 1218 | 21(1.7) | 71 | 0.55(0.32, 0.93) | 0.027 | 0.211 | 0.56(0.33, 0.95) | 0.032 | 0.249 |
| MPV Q2 (＞7.20-≤7.60fL) | 1067 | 45(4.2) | 973 | 22(2.3) | 52 | 0.53(0.32, 0.88) | 0.015 |  | 0.51(0.30, 0.85) | 0.010 |  |
| MPV Q3(＞7.60fL-≤8.0fL) | 784 | 20(2.6) | 888 | 26(2.9) | - | 1.15(0.64, 2.06) | 0.644 |  | 1.05(0.58, 1.89) | 0.868 |  |
| MPV Q4 (＞8.0fL) | 976 | 30(3.1) | 1018 | 23(2.3) | 125 | 0.73(0.42, 1.25) | 0.251 |  | 0.79(0.45, 1.37) | 0.399 |  |
| TT |  |  |  |  |  |  |  |  |  |  |  |
| Total stroke |  |  |  |  |  |  |  |  |  |  |  |
| MPV Q1 (≤7.20fL) | 446 | 15(3.4) | 467 | 15(3.2) | 500 | 0.95(0.47, 1.95) | 0.892 | 0.261 | 0.95(0.46, 1.99) | 0.898 | 0.271 |
| MPV Q2 (＞7.20fL-≤7.60fL) | 400 | 16(4.0) | 350 | 15(4.3) | - | 1.08(0.53, 2.18) | 0.831 |  | 1.03(0.50, 2.09) | 0.946 |  |
| MPV Q3(＞7.60fL-≤8.0fL) | 296 | 12(4.1) | 314 | 13(4.1) | - | 1.04(0.47, 2.27) | 0.930 |  | 1.17(0.53, 2.61) | 0.694 |  |
| MPV Q4 (＞8.0fL) | 365 | 23(6.3) | 374 | 13(3.5) | 36 | 0.55(0.28, 1.09) | 0.086 |  | 0.57(0.28, 1.13) | 0.108 |  |
| Ischemic stroke |  |  |  |  |  |  |  |  |  |  |  |
| MPV Q1 (≤7.20fL) | 446 | 12(2.7) | 467 | 13(2.8) | - | 1.03(0.47, 2.26) | 0.938 | 0.414 | 1.09(0.49, 2.45) | 0.829 | 0.561 |
| MPV Q2 (＞7.20fL-≤7.60fL) | 400 | 14(3.5) | 350 | 12(3.4) | 1000 | 0.99(0.46, 2.13) | 0.973 |  | 0.92(0.42, 2.01) | 0.829 |  |
| MPV Q3(＞7.60fL-≤8.0fL) | 296 | 10(3.4) | 314 | 13(4.1) | - | 1.25(0.55, 2.84) | 0.602 |  | 1.49(0.64, 3.48) | 0.361 |  |
| MPV Q4 (＞8.0fL) | 365 | 20(5.5) | 374 | 13(3.5) | 50 | 0.64(0.32, 1.28) | 0.203 |  | 0.67(0.33, 1.37) | 0.276 |  |
| *MTHFR* C677T Genotype Subgroups |  |  |  |  |  |  |  |  |  |  |  |
| CC&CT |  |  |  |  |  |  |  |  |  |  |  |
| Total stroke |  |  |  |  |  |  |  |  |  |  |  |
| PDW Q1 (≤15.30%) | 1176 | 43(3.7) | 1088 | 28(2.6) | 91 | 0.70(0.44, 1.13) | 0.144 | 0.892 | 0.78(0.48, 1.26) | 0.304 | 0.808 |
| PDW Q2 (＞15.30%-≤15.50%) | 1108 | 39(3.5) | 1141 | 30(2.6) | 111 | 0.74(0.46, 1.19) | 0.217 |  | 0.68(0.42, 1.10) | 0.113 |  |
| PDW Q3 (＞15.50%-≤15.70%) | 963 | 37(3.8) | 962 | 27(2.8) | 100 | 0.73(0.44, 1.19) | 0.207 |  | 0.75(0.45, 1.23) | 0.251 |  |
| PDW Q4 (＞15.70%) | 829 | 34(4.1) | 906 | 25(2.8) | 77 | 0.67(0.40, 1.11) | 0.121 |  | 0.66(0.39, 1.12) | 0.123 |  |
| Ischemic stroke |  |  |  |  |  |  |  |  |  |  |  |
| PDW Q1 (≤15.30%) | 1176 | 37(3.1) | 1088 | 19(1.7) | 71 | 0.55(0.32, 0.96) | 0.036 | 0.548 | 0.60(0.35, 1.05) | 0.076 | 0.622 |
| PDW Q2 (＞15.30%-≤15.50%) | 1108 | 36(3.2) | 1141 | 26(2.3) | 111 | 0.70(0.42, 1.15) | 0.158 |  | 0.63(0.38, 1.05) | 0.078 |  |
| PDW Q3 (＞15.50%-≤15.70%) | 963 | 32(3.3) | 962 | 25(2.6) | 142 | 0.78(0.46, 1.31) | 0.349 |  | 0.80(0.47, 1.35) | 0.396 |  |
| PDW Q4 (＞15.70%) | 829 | 29(3.5) | 906 | 22(2.4) | 90 | 0.69(0.39, 1.20) | 0.183 |  | 0.67(0.38, 1.18) | 0.167 |  |
| TT |  |  |  |  |  |  |  |  |  |  |  |
| Total stroke |  |  |  |  |  |  |  |  |  |  |  |
| PDW Q1 (≤15.30%) | 408 | 15(3.7) | 424 | 9(2.1) | 63 | 0.57(0.25, 1.31) | 0.188 | 0.216 | 0.62(0.27, 1.44) | 0.268 | 0.167 |
| PDW Q2 (＞15.30%-≤15.50%) | 423 | 10(2.4) | 400 | 19(4.8) | - | 2.04(0.95, 4.39) | 0.068 |  | 2.31(1.06, 5.04) | 0.035 |  |
| PDW Q3 (＞15.50%-≤15.70%) | 333 | 15(4.5) | 335 | 17(5.1) | - | 1.13(0.56, 2.25) | 0.740 |  | 1.32(0.65, 2.67) | 0.450 |  |
| PDW Q4 (＞15.70%) | 343 | 26(7.6) | 346 | 11(3.2) | 23 | 0.42(0.21, 0.86) | 0.017 |  | 0.41(0.20, 0.85) | 0.016 |  |
| Ischemic stroke |  |  |  |  |  |  |  |  |  |  |  |
| PDW Q1 (≤15.30%) | 408 | 11(2.7) | 424 | 8(1.9) | 125 | 0.70(0.28, 1.73) | 0.435 | 0.245 | 0.72(0.29, 1.83) | 0.496 | 0.203 |
| PDW Q2 (＞15.30%-≤15.50%) | 423 | 10(2.4) | 400 | 16(4.0) | - | 1.72(0.78, 3.79) | 0.178 |  | 1.90(0.85, 4.26) | 0.119 |  |
| PDW Q3 (＞15.50%-≤15.70%) | 333 | 12(3.6) | 335 | 16(4.8) | - | 1.32(0.63, 2.80) | 0.462 |  | 1.54(0.72, 3.30) | 0.269 |  |
| PDW Q4 (＞15.70%) | 343 | 23(6.7) | 346 | 11(3.2) | 28 | 0.48(0.23, 0.98) | 0.044 |  | 0.47(0.23, 0.98) | 0.043 |  |

Adjusted model adjusted for: age, sex, smoking status, body mass index, systolic blood pressure, diastolic blood pressure, total cholesterol, triglycerides, high-density lipoprotein cholesterol, total homocysteine, creatinine, and blood glucose. Enalapril group is the reference group. *p value for interaction test: 2-way interaction of PCT (Q1 vs. Q2-Q4)/PLT(Q1-Q4)/MPV(Q1-Q4)/PDW(Q1-Q4) and treatment groups (enalapril vs. enalapril folic acid) on first stroke. 412 cases excluded due to missing baseline PLT data, therefore the total number of PLT cases used in models is 10773. CI, confidence interval; HR, hazard ratio; MPV, mean platelet volume; *MTHFR,* methylenetetrahydrofolate reductase; NNT, number needed to treat; PDW, platelet distribution width; PLT, platelet count; Q, quartile.
